# Supplementary material for: A fault-intrusion-tolerant system and deadline-aware algorithm for scheduling scientific workflow in the cloud
Source: PeerJ Comput Sci. 2021 Nov 2;7:e747. doi: 10.7717/peerj-cs.747 (PMC8576554; doi:10.7717/peerj-cs.747)
Supplement: Supplemental Information 6 [file peerj-cs-07-747-s006.zip › Myproject1.1/docs/org/workflowsim/scheduler/package-summary.html]

org.workflowsim.scheduler


JavaScript is disabled on your browser.


- Overview
- Package
- Class
- Use
- Tree
- Deprecated
- Index
- Help

- Prev Package
- Next Package

- Frames
- No Frames

- All Classes

# Package org.workflowsim.scheduler

- Interface Summary

  | Interface | Description |
  | --- | --- |
  |  |  |
  | --- | --- |
  | SchedulerInterface | The Scheduler interface |
- Class Summary

  | Class | Description |
  | --- | --- |
  |  |  |
  | --- | --- |
  | BaseScheduler | The base scheduler has implemented the basic features. |
  | DataAwareScheduler | Data aware algorithm. |
  | FCFSScheduler | The FCFS algorithm. |
  | MaxMinScheduler | MaxMin algorithm. |
  | MCTScheduler | MCT algorithm |
  | MinMinScheduler | MinMin algorithm. |
  | StaticScheduler | Static algorithm. |

- Overview
- Package
- Class
- Use
- Tree
- Deprecated
- Index
- Help

- Prev Package
- Next Package

- Frames
- No Frames

- All Classes
